# The Effects of Transdermally Delivered Oleanolic Acid on Malaria Parasites and Blood Glucose Homeostasis in *P*. *berghei*-Infected Male Sprague-Dawley Rats

**DOI:** 10.1371/journal.pone.0167132

**Published:** 2016-12-01

**Authors:** Happiness P. Sibiya, Musa V. Mabandla, Cephas T. Musabayane

**Affiliations:** School of Laboratory Medicine and Medical Sciences, University of KwaZulu-Natal, Durban, South Africa; Instituto Oswaldo Cruz, BRAZIL

## Abstract

The present study investigated the effects of transdermally delivered oleanolic acid (OA) monotherapy and in combination with chloroquine (CHQ) on malaria parasites and glucose homeostasis of *P*. *berghei*-infected male Sprague-Dawley rats. Oral glucose test (OGT) responses to OA-pectin patch and CHQ-OA combination matrix patch were monitored in non-infected and infected rats. To evaluate the short-term effects of treatment, percentage parasitaemia, blood glucose, glycogen and plasma insulin were monitored in separate groups of animals treated with either OA-patch monotherapy or CHQ-OA combination pectin patch over a 21-days period. Animals treated with drug-free pectin and CHQ acted as untreated and treated positive controls, respectively. Infected control rats exhibited significantly increased parasitaemia which was accompanied by hypoglycaemia. Both OA monotherapy and CHQ-OA combination therapy reduced and cleared the malaria parasites within a period of 4 and 3 days, respectively. Compared to respective controls groups, OGT responses of animals treated with OA monotherapy or CHQ-OA combination therapy exhibited lower blood glucose levels at all time points. A once-off transdermal application of OA-patch or CHQ-OA combination patch significantly improved blood glucose concentrations inducing any changes in insulin concentration. Transdermal OA used as a monotherapy or in combination with CHQ is able to clear and reduce the malaria parasites within a shorter period of time without eliciting any adverse effects on glucose homeostasis of *P*. *berghei*-infected rats.

## Introduction

Malaria continues to be a health problem globally, an estimated 3.3 billion people are at risk of developing the disease [1]. According to the World Health Organization (WHO), 438 000 deaths were reported in 2015, with 90% of these cases occurring in the African region [1]. Reports indicated that the high parasitaemia resulting from infection is associated with the derangement of some physiological systems including the impairment of the glucose homeostasis, acute renal failure and anaemia [2–5]. Severe hypoglycaemia has been reported in African children infected with *Plasmodium falciparum*. This decrease in blood glucose has been attributed to increased utilisation of the host’s glucose stores by the malaria parasites [3]. Furthermore, studies have shown that the depletion of vital gluconeogenic substrates, such as thiamine, by the malaria parasites plays a major role in the reported malaria-associated hypoglycaemia [6]. This hypoglycaemia may be exacerbated by some antimalarial drugs, including quinine and CHQ [7–8].

The widespread resistance of *Plasmodium* parasites to commonly used anti-malarial drugs, such as mefloquine and CHQ, has forced countries to review and deploy new anti-malarial drug policies. The current treatment for malaria involves the use of artemisinin-derived combination therapies (ACTs) [9]. ACTs have and still remain the first-line treatments for uncomplicated *Plasmodium falciparum* malaria in many malaria-endemic areas [9–10]. The use of these combination therapies has resulted in a significant reduction in malaria-related mortalities and overall transmission of the disease over the past decade [11–12]. Despite their success in reducing malaria mortality rates and lowering the rate at which resistance occurs, therapeutic failure of ACTs has been reported in some areas, including Southeast Asia [13–15]. The emergence of ACT-resistant strains of *Plasmodium falciparum* poses a major threat in many endemic areas, such as Southeast Asia, India and Africa. Hence, there is an urgent need to search and develop novel antimalarial agents which are able to eliminate the malaria parasites from systemic circulation within a short time period and ameliorate malaria-related complications. *Syzygium aromaticum (S*. *aromaticum*) [Mirtaceae family] commonly known as clove has been widely used in folkloric medicine for the treatment of a variety of ailments. Reports indicate that *S*. *aromaticum*-derived oleanolic acid (OA) and maslinic acid (MA) possesses anti-inflammatory, antibacterial, antioxidant, and hepatoprotective properties [16–19]. We, therefore, speculate that these plant extracts may exert antiplasmodial effects against *P*. *berghei* parasites *in vivo*. Studies have shown that these triterpenes are of low solubility when administered orally, which ultimately affects their bioavailability [20–21]. We have reported on the ability of transdermal CHQ and insulin matrix-pectin patch to provide sustained controlled release of CHQ and insulin over a period of time, respectively [22–24]. Against this background, the current study evaluated the effects of transdermally delivered OA and CHQ-OA combination on *P*. *berghei* parasites and glucose homeostasis in male Sprague-Dawley rats.

## Materials and Methods

### Drug and chemicals

All drugs and chemicals which were of analytical grade quality were sourced from standard pharmaceutical suppliers. Chloroquine diphosphate (C_18_H_26_CIN_3_∙2H_3_PO_4_), anthrone reagent, sigmacote, dimethyl sulphoxide (DMSO), Giemsa stain and May-Grunwald solution (Sigma-Aldrich Chemical Company, St Louis, Missouri, USA); calcium chloride (CaCl_2_), potassium hydroxide (KOH), sodium sulphate (Na_2_SO_4_), sodium hydroxide (NaOH), potassium dihydrogen phosphate (KH_2_PO_4_) and 95% ethanol (C_2_H_5_OH) (Merck Chemicals (PTY) LTD, Johannesburg, South Africa); diethyl ether (C_4_H_10_O) (NT Laboratory Supplies (PTY) LTD, Johannesburg, South Africa); sulphuric acid (H_2_SO_4_) (BDH Chemicals LTD, Poole, Dorset, England), halothane (Fluorothane^®^, AstraZeneca Pharmaceuticals (PTY) LTD, Johannesburg, South Africa) and insulin ELISA kit (Mecrodia AB, Uppsala, Sweden). *S*. *aromaticum* dried flower buds were purchased from Kies Supermarket (Reservoir Hills, Durban, South Africa)

### Isolation of OA

OA was isolated from *S*. *aromaticum* [(Linnaeus) Merrill & Perry] [Myrtaceae] (cloves) flower buds using a standard protocol that has been validated in our laboratory with slight modifications [25–26]. Briefly, air-dried *S*. *aromaticum* flower buds (500 g) were sequentially extracted twice at 24-hour intervals at room temperature with 1 L dichloromethane (DCM), and ethyl acetate (720 mL) on each occasion. Removal of the solvent from the extract under reduced pressure at 55±1°C using a rotary evaporator yielded dichloromethane solubles (DCMS, 63 g) and ethyl acetate solubles (EAS, 85 g). The EAS containing mixtures of oleanolic/ursolic acid was purified by silica gel 60 column chromatography with a hexane: ethyl acetate 9:1, 8:2 solvent system increasing polarity. OA was yielded. The structure of OA was confirmed by spectroscopic analysis using 1D and 2D, ^1^H and ^13^C Nuclear Magnetic Resonance (NMR) techniques.

### Patch preparation

Amidated pectin hydrogel OA and CHQ-OA matrix patches were prepared using a well-established protocol by Musabayane *et al*., (2003) with slight modifications [22]. Briefly, amidated low methoxyl pectin was dissolved in deionized water (4.4 g/110 mL) followed by adding OA (1.44 g dissolved in DMSO) (Sigma-Aldrich Chemical Company, Missouri, St Louis, USA). However, to prepare the CHQ-OA combination patch, CHQ (5 g) and OA, (1.44 g dissolved in DMSO) were added together in one beaker, followed by agitation for 30 minutes. Subsequently, eucalyptus oil (1.65 mL, Barrs Pharmaceutical Industries cc, Cape Town, South Africa) and vitamin E (1.65 mL, Pharmacare Ltd, Johannesburg, South Africa) were added to the mixture and spun for another 1 hour 30 minutes. Following this, an aliquot (11 mL) was transferred to a petri dish with a known diameter and frozen at -4°C for 18 hours. After freezing, 1.5 mL of a 2% CaCl_2_ solution was added to allow for cross-linking and formation of the matrix patch. The patches were then stored in a refrigerator at 4°C until use.

### Animals

Male Sprague-Dawley rats (90-120g body weight) bred and maintained at the Biomedical Research Unit, University of KwaZulu-Natal were used. The animals had free access to standard rat chow (Meadows Feeds, Pietermaritzburg, South Africa) and water, with a 12-hour light/12 h-hour dark cycle. The animals were monitored twice daily at 8h00 and 17h00. Humane endpoints were also monitored daily and the animals were assessed on the following: alertness upon handling, mobility, type of breathing (rapid, shallow, laboured or normal) change in body weight, haematocrit, % baseline weight change, food and water consumption. Animals that stopped eating, drinking water or lost more than 20% of baseline weight at any time point were euthanised by exposing to halothane (100 mg/kg) for 3 minutes via a gas anaesthetic chamber. Untreated *P*. *berghei*-infected control animals were closely monitored, for ethical reasons the infected control animals were sacrificed on day 12 of the 21 days study to alleviating any pain and distress. The animals were sacrificed by exposing to halothane (100 mg/kg) for 3 minutes via a gas anaesthetic chamber. All animal experimentation was reviewed and approved by the Animal Ethics Committee of the University of KwaZulu-Natal (**095/14/Animal**).

### Induction of malaria

Malaria was induced in male Sprague-Dawley rats with a single intraperitoneal injection of *P*. *berghei* parasitized red blood cells (10^5^) [27]. Control animals were injected with phosphate buffered saline vehicle. Successful malaria induction was confirmed by microscopic examination of Giemsa-stained thin smears of the rat tail blood. Percentage parasitaemia of greater than or equal to 20% was considered as a stable malaria state before commencing any experimental procedures.

### Application of the hydrogel matrix patch

Rats were shaved on the dorsal region of the neck using the Oster Golden A5 heavy duty single-speed animal clipper (Oster Professional products, McMinnville, Tennessee, United States) 24 hours prior to the application of hydrogel matrix patches. The dermal patches were secured in place with adhesive hydrofilm (Hartman-Congo Inc, Rock Hill, South Carolina, USA) and rat jackets (Braintree, Scientific, Inc, Braintree, Massachusetts, USA) which were adjusted according to the size of the animal.

### Acute studies

OGT responses following transdermal application of either OA-pectin patches (34 mg/kg) or CHQ-OA (56 /34 mg/kg) pectin patches and oral OA (160 mg/kg) were evaluated in separate groups of non-infected and *P*. *berghei*-infected rats as previously described by Musabayane *et al* [28]. Briefly, separate groups of non-infected and infected rats were fasted overnight (18 hours). OGT responses were monitored over a 4-hour period. Animals treated with drug-free pectin and CHQ (30 mg/kg, p.o) acted as untreated and treated positive control animals. Blood glucose was measured using a glucometer (OneTouch select glucometer, Lifescan, Mosta, Malta, United Kingdom) at 15 minutes intervals for the first hour and then hourly for the subsequent 4 hours after glucose loading (0.86 mg/kg). Blood samples were collected by cardiac puncture into pre-cooled heparinized tubes after 4 hours from all groups for plasma insulin measurements.

### Short-term studies

To evaluate the short-term effects of transdermally delivered OA (34 mg/kg) and a CHQ-OA (56/34 mg/kg) combination, patches were topically applied onto the shaved skin area on the back of the neck. The patches were only applied once at 9h00 on day 7 of the treatment period. Oral CHQ (30 mg/kg) and OA (160 mg/kg) were administered twice daily for 5 consecutive days. Animals treated with drug-free pectin and CHQ acted as negative and positive controls, respectively. Blood glucose, body weights, amounts of water and food consumed were measured in control and treated animals at 09h00 every 3^rd^ day during the pre-treatment and post-treatment periods. However, following the application of dermal patches, parameters were monitored on selected days (9 and 12). Blood glucose concentrations were measured using blood glucose testing strips, (OneTouch select glucometer, Lifescan,Mosta, Malta, United Kingdom).

### Parasitaemia monitoring

Daily malaria parasite density was monitored by microscopic counting of Giemsa-stained thin blood smears of *P*. *berghei*-infected animals throughout the 21-day experimental period. The blood smears were collected 24 hours after treatment at 9h00. Parasitaemia was scored on Giemsa-stained tail-blood films under a microscope (Olympus cooperation, Tokyo, Japan) with a x50–x100 oil immersion objective (Olympus cooperation, Tokyo, Japan).

### Terminal studies

On selected days (days 0, 7, 9, 12 and 21), separate groups of animals were sacrificed by exposing to halothane (100 mg/kg) for 3 minutes via a gas anaesthetic chamber. Blood was collected by cardiac puncture into individual pre-cooled heparinized tubes. Separated plasma was analysed for insulin concentrations. Thereafter, the liver and muscle tissues were removed snap frozen in liquid nitrogen and stored in a BioUltra freezer (Snijders Scientific, Tilburg, Netherlands) at -80°C until use. The liver and muscle were analysed for glycogen content.

### Insulin concentrations measurements

Plasma insulin concentrations were determined in blood samples of separate groups of non- infected and infected animals following treatment with various patch formulations. The assays were performed on ultrasensitive rat insulin ELISA kit (Mercodia AB, Uppsala, Sweden). The lower limit of detection was 0.020 μg/L. The intra- and interassay analytical coefficients of variation ranged from 2.0 to 2.9% and from 3.5 to 4.8% respectively.

### Glycogen measurements

Glycogen concentrations were determined as previously described by Khathi *et al* [29]. Liver and muscle tissue samples (1–1.5 g) were homogenised in 2 mL of 30% KOH solution and digested at 100°C for 30 minutes. The homogenate was then cooled in ice-saturated sodium sulphate. After cooling, glycogen was precipitated with ethanol, pelleted, washed, and dissolved in deionized water. Glycogen standards (10–2000 mg/L) were also prepared using glycogen powder. The glycogen concentration was determined by its reaction with the anthrone reagent (2 g anthrone/L of 95% (v/v) H2SO4) after which absorbance was measured at 620 nm using a NovaspecII spectrophotometer (Biochrom Ltd., Cambridge, UK).

### Skin histology

The effects of dermal-hydrogel matrix patch applications on the skin were evaluated using histological analysis. Following collection, the skin samples were fixed in formalin solution (10%). This was followed by rehydration in decreasing grades of ethanol and embedding in paraffin wax. The samples were then sectioned using a microm rotary microtome (Robert-Bosch-Straβe, Walldorf, Baden-Württemberg, Germany). Subsequently, the sections were stained with haematoxylin and eosin (H and E) followed by dehydration in increasing grades of ethanol and cleared in xylene. The sections were viewed and captured using Leica light microscope (Leica Biosystems Peterborough Limited, Peterborough, Berkshire, UK).

### Statistical analysis

All data were expressed as means ± standard error of means (S.E.M.). Statistical comparison of the differences between the control means and experimental groups was performed with GraphPadInStat Software (version 5.00, GraphPad Software, San Diego, California, USA), using one-way analysis of variance (ANOVA), followed by Tukey-Kramer multiple comparison test. A value of p˂0.05 was considered significant.

## Results

### Structural elucidation of OA

The isolated OA was identified by 1H NMR and 13C NMR (1D and 2D). This data compared with those reported in the literature [25]. The purity of the plant-derived OA was approximately 98% and the percentage yield varied from 0.02% to 0.03%.

### Effect of treatments on parasitaemia

**Fig 1:**
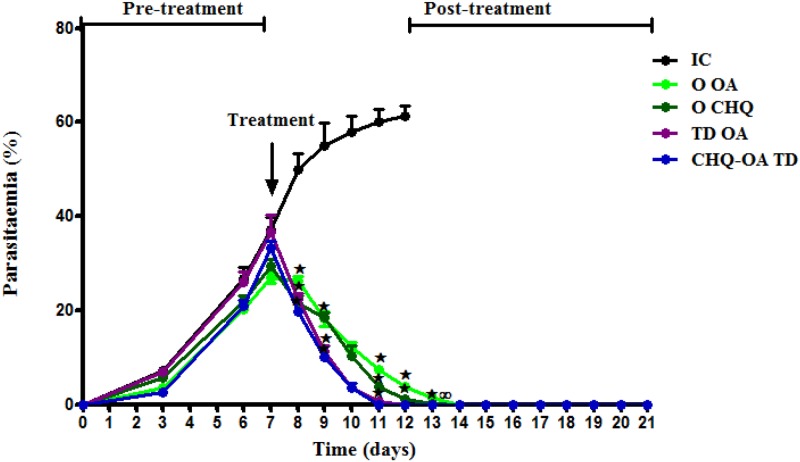
The effects of oral administration of OA (O OA), transdermal application of OA (TD OA) and CHQ-OA (TD CHQ-OA) pectin matrix patches on percentage parasitaemia in *P*. *berghei*-infected rats. Values are presented as means, and vertical bars indicate SEM of means (day 0, n = 30; day 7, n = 24; day 9, n = 18; day 12, n = 12; day 21, n = 6). ^⋆^p˂0.05 by comparison with control animals.^∞^p˂0.05 by comparison with oral CHQ treated animals.

The infected untreated control showed a continuous increase in percentage parasitaemia reaching 61.2 ± 2.3% by day 12 post infection. These control animals were sacrificed on day 12 due to ethical reasons. A once-off topical application of OA and CHQ-OA dermal patches significantly decreased percentage parasitaemia to undetectable levels by day 12 and 11 respectively. Oral administration of either OA or CHQ twice daily, reduced and cleared all residual parasites from the systemic circulation by day 14 (Fig 1).

### OGT responses

**Fig 2:**
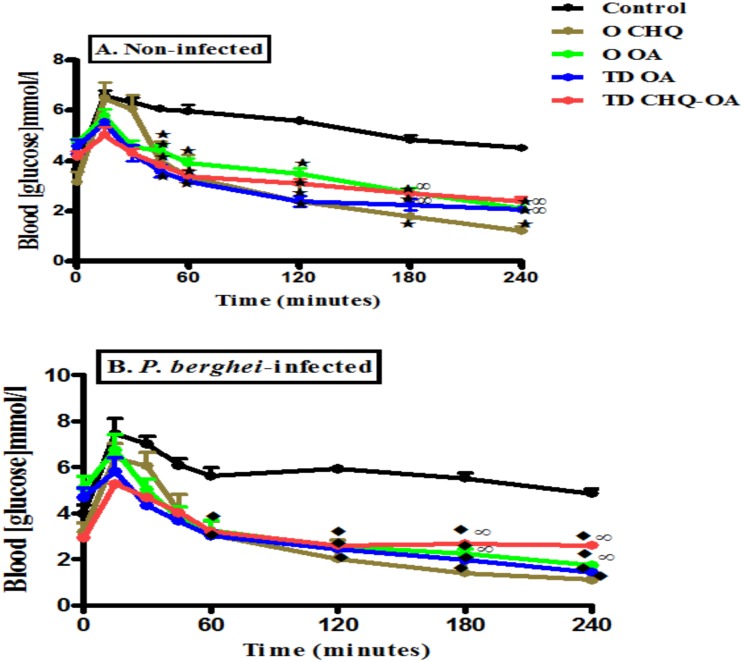
Comparisons of OGT responses in non-infected [A] and *P*. *berghei*-infected rats [B] to different antimalarial formulations with respective control animals. Values are presented as means, and vertical bars indicate SEM of means (n = 6 in each treatment group). ^⋆^p˂0.05 by comparison with non-infected control animals (NIC), ^♦^p˂0.05 by comparison with infected control (IC) animals and ^∞^p˂0.05 by comparison with CHQ treated animals (O CHQ).

Following a glucose load, there was a significant increase in blood glucose concentrations of non-infected (NIC) and infected animals (IC) (Fig 2). A once-off topical application of OA matrix patch (TD OA), significantly decreased glucose levels of non-infected and infected animals, a similar trend was observed in animals treated with oral OA (O OA). Transdermal administration of OA in combination with CHQ (TD CHQ-OA) reduced blood glucose of non-infected and infected animals throughout the 4-hour period. Oral administration of CHQ (O CHQ) also decreased blood glucose concentrations of non-infected and *P*. *berghei*-infected animals reaching hypoglycaemic values by the end of the experimental period.

### Short-term effects of OA on blood glucose

**Fig 3:**
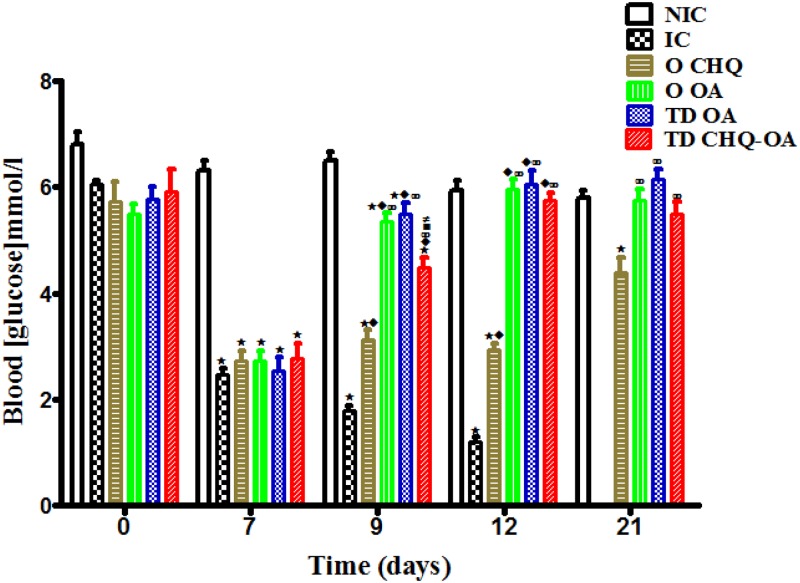
Comparison of the effects of oral administration of OA (O OA), transdermal application of OA (TD OA) and CHQ-OA combination (TD CHQ-OA) pectin matrix patches on blood glucose concentrations with those of respective controls and a standard drug CHQ (O CHQ). Values are presented as means, and vertical bars indicate SEM of means (n = 30 in each treatment group). ^⋆^p˂0.05 by comparison with non-infected control animals (NIC), ^♦^p˂0.05 by comparison with infected control animals (IC), ^∞^p˂0.05 by comparison with CHQ treated animals (O CHQ), ^■^p˂0.05 by comparison with oral OA treated animals (O OA), ^#^p˂0.05 by comparison with transdermal OA treated animals (TD OA) and ^●^p˂0.05 by comparison with CHQ-OA **treated** animals (TD CHQ-OA).

Infected control (IC) animals exhibited reduced blood glucose concentrations in comparison to the non-infected control (NIC) (Fig 3). Topical application of OA- pectin patches (TD OA) significantly increased blood concentrations of infected animals (Fig 3). Following transdermally delivery of CHQ-OA combination (TD CHQ-OA) to infected animals there was a significant increase in blood glucose levels of these animals when compared with respective controls (Fig 3). However, the blood glucose concentrations of animals treated with combination therapy were slightly lower when compared to those treated with transdermal OA and oral monotherapy (Fig 3). Oral CHQ treated animals exhibited significantly reduced blood glucose in comparison to other treatment groups throughout the treatment period (Fig 3)

### Effect of treatments on glycogen content

Infected control (IC) animals showed significantly reduced hepatic and muscle glycogen content in comparison to the non-infected control animals (Table 1). Transdermal (TD OA) and oral OA (O OA) treatment improved hepatic glycogen content in malaria-infected animals in comparison with infected control animals. Similar responses were observed in animals treated with CHQ-OA combination (TD CHQ-OA) pectin patch, where hepatic glycogen concentrations reached values comparable to those of the non-infected control animals (NIC). However, on day 12 animals treated with oral CHQ (O CHQ) showed increased hepatic glycogen concentrations relative to the non-infected controls animals (NIC).

**Table 1:** The effect of transdermally delivered OA (TD OA) alone and in combination with CHQ (CHQ-OA) on hepatic and gastrocnemius glycogen concentrations of *P*. *berghei*-infected rats in comparison with control animals (NIC and IC).

Group/treatment	Time (Days)	Glycogen g 100^−1^ g tissue	
		Hepatic	Muscle
NIC	9	25.6 ± 0.6	52.0 ± 0.9
	12	26.3 ± 0.9	52.9 ± 0.8
	21	27.4 ± 0.7	53.4 ± 0.9
IC	9	17.0 ± 0.4	52.5 ± 0.8
	12	16.0 ± 0.3	50.9 ± 0.5
O CHQ 30	9	48.2 ± 1.0^⋆^	63.0 ± 0.5^⋆^^♦^
	12	51.5 ± 1.1^⋆^	65.2 ± 0.6^⋆^^♦^
	21	26.8 ± 0.6	55.1± 0.4
O OA	9	22.7 ± 0.9^♦^^∞^	49.4 ± 0.9^∞^
	12	21.3 ± 1.0^♦^^∞^	39.0 ± 0.6^∞^
	21	20.3 ± 0.6^∞^	55.0 ± 0.4
TD OA	9	28.1 ± 0.6^♦^^∞^	55.4 ± 0.8^∞^
	12	28.0 ± 0.9^♦^^∞^	42.3 ± 0.6^∞^
	21	19.2 ± 0.5^∞^	54.0 ±0.6^∞^
TD CHQ-OA	9	24.7 ± 0.4^♦^^∞^	51.3 ± 0.5^∞^
	12	28.6 ± 0.7^♦^^∞^	40.3 ± 0.5^∞^
	21	23.0 ± 0.6^♦^	44.7 ± 0.8^∞^

(n = 30 in each treatment group). Values are presented as means ± SEM

^⋆^p<0.05 by comparison with the non- infected control (NIC) animals

^♦^p<0.05 by comparison with the infected control (IC) animals

^**∞**^p<0.05 by comparison with the CHQ-treated animals

### Effects of treatments on plasma insulin secretion

**Fig 4:**
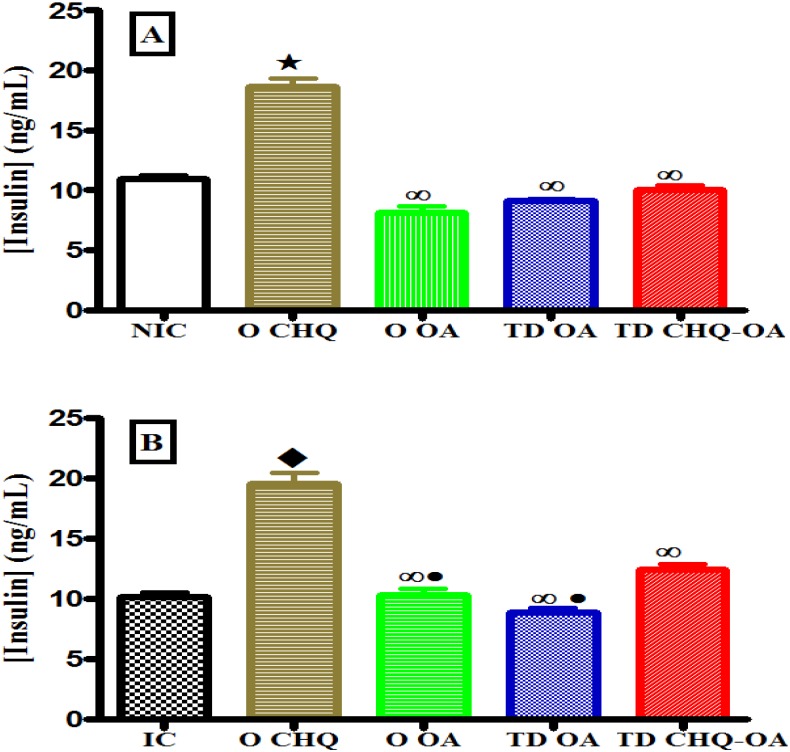
Acute effects of different antimalarial formulations on plasma insulin concentrations in non-infected (A) and *P*. *berghei*-infected (B) rats following a 4-hour glucose tolerance test. **The blood samples were collected 4 hours after treatment.** Values are presented as means, and vertical bars indicate SEM of means (n = 6 in each treatment group).^⋆^p˂0.05 by comparison with non-infected control (NIC) animals, ^♦^p˂0.05 by comparison with infected control (IC) animals, ^∞^p˂0.05 by comparison with CHQ (O CHQ) treated animals.

Acute administration of OA orally (O OA) or transdermally (TD OA) had no effects on terminal plasma insulin concentrations of non-infected (Fig 4A) and *P*. *berghei*-infected (Fig 4B) animals. Transdermal application of CHQ-OA containing patches had no significant effect on plasma insulin concentrations in infected control animals. Oral administration of CHQ significantly increased plasma insulin concentrations of non-infected and infected in comparison when compared with other treatment groups (Fig 4A and 4B).

**Fig 5:**
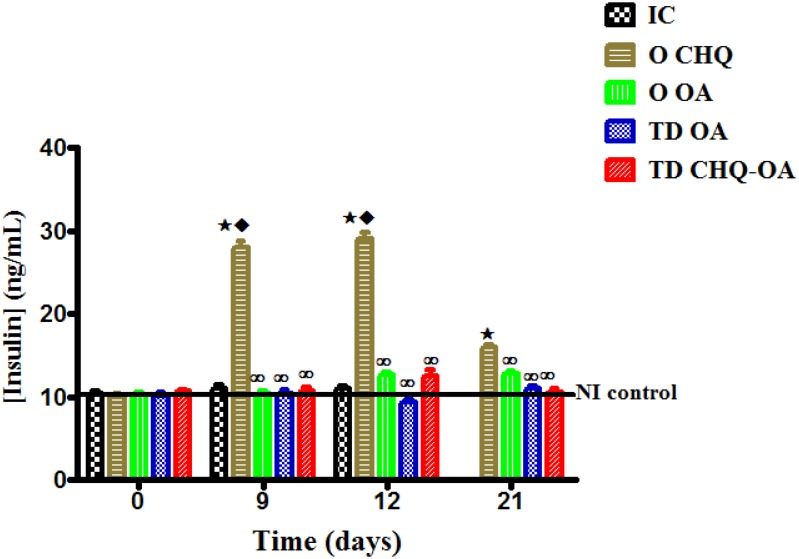
Comparison of the short-term effects of oral administration of OA (O OA), transdermal application of OA (TD OA) and CHQ-OA (TD CHQ-OA) pectin matrix patches with respective controls and a standard drug CHQ (D) on plasma insulin concentrations in *P*. *berghei*-infected rats with non-infected (NIC) and infected control (IC) animals. Values are presented as means, and vertical bars indicate SEM of means (n = 30 in each treatment group).^⋆^p˂0.05 by comparison with non-infected control (NIC) animals, ^♦^p˂0.05 by comparison with infected control (IC) animals, ^∞^p˂0.05 by comparison with CHQ (O CHQ) treated animals.

Short-term administration of OA either orally (O OA) or transdermally (TD OA) had no effect on plasma insulin concentrations throughout the experimental period. There were no significant changes in plasma insulin concentrations following a once-off transdermal application of the CHQ-OA combination (TD CHQ-OA) patch (Fig 5). Oral administration of CHQ twice daily (OCHQ) significantly (p<0.05) increased plasma insulin concentrations of *P*. *berghei*- infected (NIC) (Fig 5).

### Food and water intake

Food and water intake of non-infected control (NIC) animals remained unchanged throughout the study, whilst the animals progressively gained weight (Table 2). When compared to non-infected control, both *P*. *berghei*-infected control (IC) and oral CHQ (O CHQ) treated animals showed a significant (p<0.05) reduction in food and water intake. This reduction in food and water intake was associated with a reduction in body weight. There were no changes in food and water intake in animals treated with either transdermal OA (TD OA) or CHQ-OA combination patch (TD CHQ-OA). However, a significant reduction in body weight during the treatment period was observed in these animals. During the post-treatment, period transdermally treated animals gained weight, reaching values comparable to the non-infected control (Table 2).

**Table 2:** The effect of transdermally delivered OA (TD OA) alone and in combination with CHQ (CHQ-OA) on body weight, food and water intake in *P*. *berghei*-infected rats in comparison to control animals (NIC and IC).

Parameter	Group	Pre-treatment	Treatment	Post-treatment
Food intake (g/100g)	NIC	11.0 ± 2.0	12.0 ± 1.0	14± 2
	IC	10.0 ± 1.0	6.0 ± 2.0^⋆^	N/A
	O CHQ	10.0 ± 1.0	4.0 ± 1.0^⋆^	11.0 ± 1.0
	O OA	10.0 ± 1.0	11.0 ± 1.0^♦^^∞^	12.0 ± 2.0
	TD OA	11.0 ± 2.0	11.6 ± 1.0^♦^^∞^	18.4 ± 3^⋆^
	TD CHQ-OA	11.0 ± 2.0	12.3 ± 2^♦^^∞^	14.7 ± 3
Water intake (mL/100g)	NIC	15.0 ± 2.0	16.0 ± 3.0	17.0± 2.0
	IC	8.0 ± 3.0	8.0 ± 2.0^⋆^	N/A
	O CHQ	8.0 ± 2.0	7.0 ± 2.0^⋆^	12.0 ± 2.0^⋆^
	O OA	12.0 ± 1.0	14.0 ± 4.0^♦^	14.0 ± 2.0
	TD OA	11.0 ± 1.0	14.0 ± 2^♦^	16.0 ± 2.0
	TD CHQ-OA	11.0 ± 1.0	15.0 ± 2^♦^	14.0 ± 2 ^⋆^
% b. wt change	NIC	8.0 ± 1.0	10.0 ± 1.0	18 ± 1.0
	IC	-6.0 ± 2.0	-4.0 ± 2^⋆^	N/A
	O CHQ	-8.0± 1.0	-5.0± 1.0^⋆^	-1.0 ± 1.0^⋆^
	O OA	-6.0 ± 1.0	-1.0 ± 1.0^⋆^^♦^	2.0 ± 1.0^⋆^^♦^
	TD OA	-5.0 ± 1.0	2.0 ± 1.0^⋆^^♦^	8.0 ± 1.0^⋆^^♦^
	TD CHQ-OA	-4.0 ± 1.0	2.0 ± 1.0^⋆^^♦^	6.0 ± 1.0^⋆^^♦^

Oral CHQ and OA were administered twice daily for 5 consecutive days, however, for transdermal delivery the patch was applied once-off at the beginning of the treatment period. (n = 30 in each treatment group). Values are presented as means ± SEM

^⋆^p<0.05 by comparison with the non- infected control (NIC) animals

^♦^p<0.05 by comparison with the infected control (IC) animals

^**∞**^ p<0.05 by comparison with the CHQ-treated animals

### Effect of dermal patches on the skin

**Fig 6:**
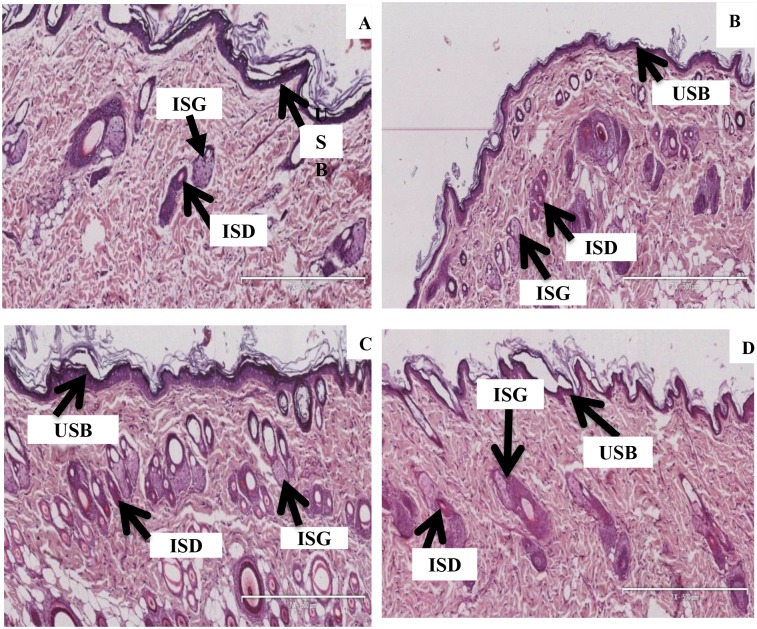
H and E stains illustrating the effects of OA-containing dermal patches on the morphology of the skin. Picture (6A) represents intact secretory ducts (ISD), uninjured stratum basale (USB) and intact sebaceous glands (ISG) of the non-infected control animals (Mag 7×500 μm). Picture 6B represents intact secretory ducts, uninjured stratum basale and intact sebaceous glands of the *P*. *berghei*-infected control (Mag 7×500 μm).Treatment with oral CHQ picture 6C (Mag 7×500 μm) and OA dermal patches picture 6D (Mag 8×500 μm) showed intact secretory ducts, uninjured stratum basale and intact sebaceous glands.

Fig 6 shows the H and E skin stained sections of untreated non-infected control (NIC), infected control (IC), *P*. *berghei*-infected rats treated with a once-off topical application of the pectin OA matrix patch (TD OA) and oral CHQ (O CHQ). Fig 6A represents intact secretory ducts (ISD), uninjured stratum basale (USB) and intact sebaceous glands (ISG) of the non-infected control animals (NIC). Photomicrograph (6B) shows *P*.*berghei*-infected control (IC) and photomicrograph (6C) shows *P*.*berghei*-infected oral CHQ treated group. Photomicrograph (6D) represents intact secretory ducts (ISD), uninjured stratum basale (USB) and intact sebaceous glands (ISG) of the *P*. *berghei* infected treated with once-off topical application of the OA pectin patch. Compared to the non-infected control animals (Fig 6A), neither inflammation nor necrosis was detected in the skin as the photomicrographs revealed preserved epidermis and dermis after the treatment period.

## Discussion

The results presented in this study show that a once-off transdermal application of OA-alone or in combination with CHQ clears the malaria parasites in *P*. *berghei*-infected rats. Furthermore, there was no relapse that occurred during the post-treatment period. This data demonstrates the ability of the OA-pectin patch to deliver sustained controlled therapeutic doses of OA which reduced and cleared the malaria parasites within a period of 4 days. Indeed, we have previously reported on the ability of transdermal systems to deliver drugs in a sustained controlled manner [23–30]. Similar to CHQ, both transdermal and orally administered OA were able to reduce parasitaemia to undetectable levels. Previous studies have reported the antiplasmodial activities of OA against CHQ *sensitive Plasmodium falciparum in vitro* [31–32]. Bero and colleagues also demonstrated the antiplasmodial properties of *Keetia leucantha*-derived OA *in vitro* [33]. However, the previous studies only demonstrate the *in vitro* effects. The data presented in the current study demonstrates for the first time, the potent antiplasmodial activity of OA against *P*. *berghei* parasites in an *in vivo*. According to Sairafianpour *et al*., OA exerts its antiplasmodial activity by incorporating into the membranes of erythrocytes and mediates the transformation of erythrocytes into stomatocytes [31]. Interestingly, the OA patch formulation showed synergistic effects when combined with CHQ, as indicated by the elimination of parasitaemia in as shorter period of time. This data provides evidence that transdermally administered OA as a monotherapy or in combination with CHQ possesses activity against *P*. *berghei in vivo*. Previous studies have shown that orally administered OA is susceptible to hepatic degradation, which ultimately results into reduced bioavailability of the drug [21–34]. As a result of this hepatic metabolism, higher drug concentrations are required to achieve therapeutic effects. This might explain the slow elimination of parasitaemia by orally administered OA, which only cleared the malaria parasites after 7 days. The dose that was used in the current study, 160 mg/kg, was selected based on preliminary studies, where 3 doses of OA were evaluated for their antimalarial activities against *P*. *berghei* parasites. The highest dose, 160 mg/kg, was the most potent of the three. When we compared the antiplasmodial effects of transdermally delivered OA at a dose of 34 mg/kg to those of oral OA at 160 mg/kg, transdermal OA was able to clear parasitaemia within a period of 4 days. This demonstrates the ability of the OA-patch formulation to bypass hepatic degradation, which may have improved the bioavailability of OA, allowing for a quicker elimination of parasitaemia. Indeed transdermal delivery systems have been reported to bypass the liver which allows for the use of lower drug concentrations and delivering drugs directly into the systemic circulation [35]. This further demonstrates the advantage of using the OA-patch over oral OA. Furthermore, the OA patch formulation showed synergistic effects when combined with CHQ, as indicated by the reduction and clearance of parasitaemia within a period of time 3 days. According to Lu *et al*., the administration of OA at a dose of 500 μmol/kg (225 mg/kg) for 10 days induced liver injury in mice, which indicates the toxic effects of this triterpene when using high doses [36]. OA doses from 225–450 mg/kg orally administered were shown to exert toxic effects in mice [36]. The doses used in this study, 160 mg/kg (oral) and 34 mg/kg (transdermal) are lower when compared to 225 mg/kg that was used in the previous study, this could minimise the possibility of adverse effects of OA. However, the toxic effects of these doses and the antiplasmodial of this triterpene against *Plasmodium falciparum in vivo* remain to be investigated.

The acute blood glucose lowering effect of the OA-pectin patch demonstrates the ability of this novel formulation to deliver OA at a sustained controlled manner. Indeed, transdermal delivery systems have been shown to offer controlled release of drugs over longer periods of time [37–38]. The acute blood glucose lowering effects of CHQ that were observed might be attributed to the CHQ-induced increase in plasma insulin concentrations. Indeed, there was a concomitant increase in plasma insulin concentrations at the end of the 4h period. These results are in agreement with previous reports that have demonstrated the ability of CHQ to lower blood glucose levels by increasing plasma insulin concentrations [39–40]. The increase in plasma insulin concentrations following oral administration of CHQ is suggestive of CHQ toxicity on glucose homeostasis.

Hypoglycaemia is an important complication of severe *Plasmodium falciparum* malaria, particularly in children and pregnant women. The ability of OA treatment to increase blood glucose concentrations in infected rats demonstrates the beneficial effects of using OA as an antiplasmodial agent. The utilisation of the host’s glucose stores by the malaria parasites has been reported to cause of hypoglycaemia in malaria infection [41].We believe that the reduction and subsequent elimination of parasitaemia by transdermal played a significant role in ameliorating hypoglycaemia. Indeed, there was a correlation between blood glucose concentrations and percentage parasitaemia at each time interval, without any changes in plasma insulin concentrations. OA has been shown to possess hepatoprotective properties [42]. The hepatoprotective properties of OA may in part play a role in improving blood glucose levels back to normoglycaemia. Unlike OA and CHQ-OA, CHQ treatment showed adverse effects on glucose homeostasis by exacerbating hypoglycaemia. These results are in agreement with previous findings which have reported the adverse effects of CHQ on glucose homeostasis [7–43]. Furthermore, the bitter taste of CHQ is believed to result in reduced food and water intake which can aggravate hypoglycaemia during treatment. The use of the CHQ-OA patch was able to avoid the bitter taste of CHQ by delivering the drug through the skin and allowed for the continuous food consumption. The continuous food intake subsequently improved the blood glucose concentrations in the infected animals.

Glycogen concentrations of animals treated with transdermal OA were comparable to those of the non-infected control animals. This data further demonstrates the ability of OA to improve glucose homeostasis without having any adverse effects in rats. The data also shows that OA-associated increase in blood glucose levels is mainly mediated through the removal of the malaria parasites from the systemic circulation. Hepatic and muscle glycogen contents were increased following treatment with oral CHQ. The increase in glycogen concentration might attribute to increased plasma insulin following treatment with CHQ. We speculate that this increase in insulin concentrations promotes glucose uptake in the liver.

Transdermal delivery offers a convenient and non-invasive alternative route of drug delivery to oral and subcutaneous injections. The skin plays a major role in the delivery of drugs via the transdermal route. Accordingly, we evaluated the effects of transdermal patch application on the skin morphology. Transdermal OA and CHQ-OA formulations did no elicit any adverse effects on the skin morphology. We have previously reported on the ability of pectin-insulin to deliver therapeutic doses of insulin without any adverse effects on the underlying tissues of the skin [23]. The presence of antioxidants such as vitamin E and eucalyptus oil in the patch formulations ensures skin protection against inflammation following application. OA and CHQ-OA patches offer a novel, non-invasive and convenient route for administration of antiplasmodial agents without exerting any negative effects on the skin.

In summary, the data presented in this study shows for the first time that a once-off transdermal administration OA alone or in combination with CHQ is able to clear the malaria parasites in *P*. *berghei*-infected animals. The antimalarial formulations presented in the current study not only provide novel alternatives treatments for malaria, but also introduce an enhanced route of administration for CHQ which might improve patient compliance. Additionally, the transdermal route also offers a convenient dosing schedule which only requires a once-off application of the patch as opposed to multiple doses that are associated with oral administration and painful intravenous injections of CHQ. OA and OA-CHQ patches provide a novel, pain-free and convenient formulations which may be may be used for the treatment of malaria.

## Supporting Information

S1 Table Percentage parasitaemia data of *P*. *berghei-infected* and treated animals with various formulations. IC- Infected control; O CHQ- Orally administered chloroquine; O OA- Orally administered oleanolic acid; TD OA- Transdermally administered oleanolic acid; TD CHQ-OA- Transdermally administered chloroquine-oleanolic acid combination.
(DOCX)

S2 Table Data obtained from non-infected and *P berghei*-infected rats following a 4h OGT responses to various treatments. IC- Infected control; O CHQ- Orally administered chloroquine; O OA- Orally administered oleanolic acid; TD OA- Transdermally administered oleanolic acid; TD CHQ-OA- Transdermally administered chloroquine-oleanolic acid combination.
(DOCX)

S3 Table Short-term effects of various treatments on blood glucose concentrations of non-infected and infected animals. IC- Infected control; O CHQ- Orally administered chloroquine; O OA- Orally administered oleanolic acid; TD OA- Transdermally administered oleanolic acid; TD CHQ-OA- Transdermally administered chloroquine-oleanolic acid combination.
(DOCX)

S4 Table Terminal plasma insulin concentrations following a 4h oral glucose tolerance tests. IC- Infected control; O CHQ- Orally administered chloroquine; O OA- Orally administered oleanolic acid; TD OA- Transdermally administered oleanolic acid; TD CHQ-OA- Transdermally administered chloroquine-oleanolic acid combination.
(DOCX)

S5 Table Short-term effects of various treatments on plasma insulin concentrations of non-infected and *P*. *berghei*-infected animals. IC- Infected control; O CHQ- Orally administered chloroquine; O OA- Orally administered oleanolic acid; TD OA- Transdermally administered oleanolic acid; TD CHQ-OA- Transdermally administered chloroquine-oleanolic acid combination.
(DOCX)

S6 Table Liver and muscle glycogen concentrations following oral administration of CHQ (O CHQ), OA (O OA) and transdermal application of OA (TD OA) and or CHQ-OA (TD CHQ-OA). IC- Infected control; O CHQ- Orally administered chloroquine; O OA- Orally administered oleanolic acid; TD OA- Transdermally administered oleanolic acid; TD CHQ-OA- Transdermally administered chloroquine-oleanolic acid combination.
(DOCX)
