# Supplementary material for: The Effects of Transdermally Delivered Oleanolic Acid on Malaria Parasites and Blood Glucose Homeostasis in P. berghei-Infected Male Sprague-Dawley Rats
Source: PLoS One. 2016 Dec 1;11(12):e0167132. doi: 10.1371/journal.pone.0167132 (PMC5132303; doi:10.1371/journal.pone.0167132)
Supplement: S1 Table — IC- Infected control; O CHQ- Orally administered chloroquine; O OA- Orally administered oleanolic acid; TD OA- Transdermally administered oleanolic acid; TD CHQ-OA- Transdermally administered chloroquine-oleanolic acid combination. (DOCX) [file pone.0167132.s001.docx]

**S1 Table 1: Percentage parasitaemia of *P. berghei-infected* and treated animals**

| Group |  | **Parasitaemia (%)** | | | | | | | | | | | | |
| --- | --- | --- | --- | --- | --- | --- | --- | --- | --- | --- | --- | --- | --- | --- |
|  | **Day 0** | **Day 3** | **Day 6** | **Day 7** | **Day 8** | **Day 9** | **Day 10** | **Day 11** | **Day 12** | **Day 13** | **Day 14** | **Day 15** | **Day 18** | **Day 21** |
| IC | 0  0  0  0  0  0 | 6,8  5,6  8,5  7,0  6,4  8,3 | 27,4  25,7  25,6  27,4  27,8  25,9 | 40,0  39,0  32,6  36,8  33,7  40,7 | 48,1  52,0  53,1  50,1  46,5  52,0 | 55,8  49,9  54,7  56,7  55,5  57,9 | 62,1  54,0  57,7  57,5  55,3  61,5 | 60,2  59,1  62,0  58,1  56,0  64,8 | 59,9  63,6  57,8  64,1  60,0  61,9 | -  -  -  -  -  - | -  -  -  -  -  - | -  -  -  -  -  - | -  -  -  -  -  - | -  -  -  -  -  - |
| O CHQ | 0  0  0  0  0  0 | 6,1  9,0  3,1  5,0  3,0  8,1 | 25,0  23,0  19,1  21,0  18,0  25,0 | 34,1  30,0  28,0  28,1  24,0  32,0 | 22,1  18,0  15,0  23,10  24,1  26,0 | 15,0  22,0  17,0  17,1  19,2  21,0 | 7,0  18,0  15,0  5,1  7,1  10,0 | 5,0  6,1  4,0  2,0  2,1  3,0 | 1,0  1,0  0,1  2,0  1,0  1,0 | 0,10  0,00  0,01  1,00  0,10  0,01 | 0,0  0,0  0,0  0,0  0,0  0,0 | 0,0  0,0  0,0  0,0  0,0  0,0 | 0,0  0,0  0,0  0,0  0,0  0,0 | 0,0  0,0  0,0  0,0  0,0  0,0 |
| O OA | 0  0  0  0  0  0 | 6,1  5,2  5,1  3,0  0,0  2,0 | 22,0  18,0  17,2  16,1  25,0  23,1 | 35,1  23,0  25,1  20,0  31,1  26,2 | 24,1  29,3  28,0  25,5  24,0  31,5 | 25,2  16,0  18,0  14,1  20,0  16,1 | 15,0  11,1  14,  9,21  14,0  10,0 | 10,1  5,1  9,0  8,0  7,0  5,1 | 6,0  3,1  6,0  3,2  3,0  2,1 | 1,1  0,0  4,1  2,0  1,0  0,0 | 0,0  0,0  0,0  0,0  0,0  0,0 | 0,0  0,0  0,0  0,0  0,0  0,0 | 0,0  0,0  0,0  0,0  0,0  0,0 | 0,0  0,0  0,0  0,0  0,0  0,0 |
| TD OA | 0  0  0  0  0  0 | 11,0  13,3  14,1  10,0  14,2  9,0 | 20,1  19,2  21,0  23,1  20,0  18,1 | 41,2  39,1  37,1  33,0  34,2  36,0 | 22,3  19,0  26,1  28,2  20,0  19,1 | 8,2  14,0  12,0  16,3  8,0  11,1 | 0,0  7,2  4,1  6,0  0,0  7,0 | 2,0  0,0  0,0  1,0  0,0  1,1 | 0,0  0,0  0,0  0,0  0,0  0,0 | 0,0  0,0  0,0  0,0  0,0  0,0 | 0,0  0,0  0,0  0,0  0,0  0,0 | 0,0  0,0  0,0  0,0  0,0  0,0 | 0,0  0,0  0,0  0,0  0,0  0,0 | 0,0  0,0  0,0  0,0  0,0  0,0 |
| TD CHQ-OA | 0  0  0  0  0  0 | 5,0  1,1  2,0  3,2  3,0  1,1 | 18,0  23,2  19,1  22,0  19,0  25,1 | 28,0  36,2  36,1  32,0  36,0  32,1 | 22,0  18,1  21,0  19,1  20,0  19,2 | 12,0  10,1  11,0  9,2  10,1  9,1 | 7,1  5,1  3,0  4,0  1,1  1,3 | 0,0  0,0  0,0  0,0  0,0  0,0 | 0,0  0,0  0,0  0,0  0,0  0,0 | 0,0  0,0  0,0  0,0  0,0  0,0 | 0,0  0,0  0,0  0,0  0,0  0,0 | 0,0  0,0  0,0  0,0  0,0  0,0 | 0,0  0,0  0,0  0,0  0,0  0,0 | 0,0  0,0  0,0  0,0  0,0  0,0 |

NIC- Non-infected control

IC- Infected control

O CHQ- Orally administered chloroquine

O OA- Orally administered oleanolic acid

TD OA- Transdermally administered oleanolic acid

TD CHQ-OA- Transdermally administered chloroquine-oleanolic acid combination
