# Supplementary material for: The Effects of Transdermally Delivered Oleanolic Acid on Malaria Parasites and Blood Glucose Homeostasis in P. berghei-Infected Male Sprague-Dawley Rats
Source: PLoS One. 2016 Dec 1;11(12):e0167132. doi: 10.1371/journal.pone.0167132 (PMC5132303; doi:10.1371/journal.pone.0167132)
Supplement: S2 Table — IC- Infected control; O CHQ- Orally administered chloroquine; O OA- Orally administered oleanolic acid; TD OA- Transdermally administered oleanolic acid; TD CHQ-OA- Transdermally administered chloroquine-oleanolic acid combination. (DOCX) [file pone.0167132.s002.docx]

**S2 Table 2: Data obtained from non-infected and *P berghei*-infected rats following a 4h OGT responses to various treatments**

| **Group** | **Blood glucose concentrations (mmol/L)** | | | | | | | | | | | | | | | |
| --- | --- | --- | --- | --- | --- | --- | --- | --- | --- | --- | --- | --- | --- | --- | --- | --- |
|  | **Non-infected** | | | | | | | | ***P. berghei*-infected** | | | | | | | |
| Time (minutes) | 0 | 15 | 30 | 45 | 60 | 120 | 180 | 240 | 0 | 15 | 30 | 45 | 60 | 120 | 180 | 240 |
| Control | 4,1  3,8  3,3  3,5  3,6  3,7 | 6,6  6,8  6,3  6,7  6,6  6,6 | 5,9  6,6  6,3  6,4  6,7  6,1 | 6,0  5,8  6,3  6,1  6,3  5,9 | 6,3  5,9  5,5  6,0  6,4  5,7 | 4,9  6,0  5,6  5,4  5,7  5,5 | 5,2  4,6  4,9  5,0  4,5  4,8 | 4,6  4,4  4,7  4,2  4,7  4,5 | 4,2  3,7  3,9  4,4  3,7  4,0 | 7,1  7,2  7,6  7,7  7,4  7,7 | 6,8  7,0  7,3  6,6  6,9  7,4 | 6,3  5,6  5,8  6,3  6,1  6,4 | 5,8  5,5  5,6  5,2  5,7  5,8 | 5,7  6,2  5,9  6,2  5,8  5,7 | 5,2  5,7  5,9  5,5  4,9  5,8 | 4,7  5,1  4,9  4,6  4,8  5,0 |
| O CHQ | 3,2  3,8  2,5  3,0  3,1  3,0 | 6,5  6,6  6,3  6,1  6,7  6,5 | 5,5  6,3  6,0  6,4  6,1  6,0 | 3,9  4,2  4,0  4,0  3,9  4,1 | 3,4  3,3  4,0  3,1  3,2  3,0 | 2,2  2,8  2,4  2,2  2,5  2,3 | 2,0  1,8  1,7  2,0  1,5  1,8 | 1,3  1,2  1,3  1,2  1,1  1,2 | 3,1  3,3  3,2  3,1  3,2  3,1 | 6,3  5,9  6,6  6,4  6,7  6,4 | 6,0  5,5  6,3  6,1  6,4  6,0 | 4,0  4,0  3,9  4,2  4,2  3,8 | 3,1  3,0  3,2  2,8  3,3  2,9 | 1,7  1,9  2,3  2,2  2,1  1,8 | 1,2  1,5  1,2  1,7  1,4  1,4 | 1,2  1,1  1,0  1,1  1,2  1,0 |
| O OA | 4,0  4,4  4,8  5,0  5,2  4,9 | 4,9  5,6  6,4  6,8  5,6  5,4 | 3,9  5,3  3,9  5,2  4,8  4,3 | 3,5  4,8  4,8  4,7  4,6  4,2 | 3,0  4,1  3,8  4,1  4,4  4,1 | 2,9  4,0  3,6  2,6  3,9  3,9 | 2,9  2,2  2,8  2,5  3,4  2,7 | 2,2  1,6  2,1  2,2  2,7  1,9 | 4,0  6,8  4,6  4,4  5,6  4,4 | 5,8  8,8  5,4  7,8  5,9  5,3 | 5,4  6,2  4,2  5,5  3,8  4,2 | 5,1  3,6  2,5  4,7  2,9  2,4 | 4,2  2,9  2,4  4,4  2,4  2,4 | 2,4  2,7  2,2  3,2  2,2  2,2 | 2,3  2,5  2,0  2,8  1,6  2,0 | 2,2  1,9  1,6  1,8  1,1  1,9 |
| TD OA | 5,0  4,1  5,4  4,6  3,4  4,9 | 5,8  5,1  6,1  5,3  5,5  5,4 | 5,1  3,6  5,0  4,7  4,2  3,3 | 3,4  3,1  4,7  3,6  3,7  3,1 | 3,0  3,1  3,4  3,0  3,6  3,0 | 2,6  2,0  1,5  2,8  2,6  2,9 | 2,5  1,9  1,2  2,1  1,9  2,8 | 1,8  1,3  1,1  1,5  1,1  1,1 | 3,3  5,5  5,3  4,9  4,4  3,3 | 5,0  8,0  6,1  4,6  5,3  5,0 | 3,8  4,4  4,9  4,4  4,1  4,1 | 2,9  4,2  3,9  3,3  3,9  2,9 | 2,6  3,9  2,7  2,9  2,9  3,9 | 2,4  3,2  2,4  2,2  2,0  2,4 | 2,0  2,3  2,0  1,9  1,6  2,0 | 1,8  1,2  2,0  1,1  1,1  2,0 |
| TD CHQ-OA | 4,8  4,3  3,6  4,2  4,0  4,3 | 5,8  5,1  4,0  5,0  5,3  5,0 | 4,5  4,4  3,8  4,0  4,4  4,8 | 4,0  4,1  3,5  3,6  4,0  3,5 | 3,8  3,9  3,3  2,5  3,8  3,6 | 3,0  3,2  3,0  2,7  3,7  3,3 | 2,6  2,5  2,8  2,4  3,2  2,6 | 2,0  2,4  2,6  2,0  3,0  2,2 | 2,8  3,3  3,0  3,1  2,4  3,0 | 5,7  5,6  5,2  5,0  4,8  5,6 | 5,0  4,4  4,8  4,8  4,4  5,0 | 4,3  3,8  4,1  4,2  3,7  4,3 | 3,1  3,8  3,0  3,3  2,8  2,8 | 2,8  2,0  2,6  3,0  2,6  2,0 | 2,8  2,8  2,4  2,8  2,5  2,4 | 2,0  2,8  3,0  2,6  2,6  2,0 |

O CHQ- Orally administered chloroquine

O OA- Orally administered oleanolic acid

TD OA- Transdermally administered oleanolic acid

TD CHQ-OA- Transdermally administered chloroquine-oleanolic acid combination
