# Supplementary material for: The Effects of Transdermally Delivered Oleanolic Acid on Malaria Parasites and Blood Glucose Homeostasis in P. berghei-Infected Male Sprague-Dawley Rats
Source: PLoS One. 2016 Dec 1;11(12):e0167132. doi: 10.1371/journal.pone.0167132 (PMC5132303; doi:10.1371/journal.pone.0167132)
Supplement: S3 Table — IC- Infected control; O CHQ- Orally administered chloroquine; O OA- Orally administered oleanolic acid; TD OA- Transdermally administered oleanolic acid; TD CHQ-OA- Transdermally administered chloroquine-oleanolic acid combination. (DOCX) [file pone.0167132.s003.docx]

**Table 3: Short-term effects of various treatments on blood glucose concentrations of non-infected and infected animals**

| **Group** | **Blood glucose concentrations (mmol/L)** | | | | | |
| --- | --- | --- | --- | --- | --- | --- |
|  | **Day 0** | **Day 3** | **Day 7** | **Day 9** | **Day 12** | **Day21** |
| NIC | 7,0  6,5  6,6  6,7  6,8  7,2 | 5,3  7,1  5,4  6,0  6,4  6,0 | 5,9  6,5  6,3  6,6  6,8  5,8 | 6,5  6,7  5,8  6,9  6,2  6,9 | 6,5  5,6  5,9  5,7  6,3  5,7 | 6,9  5,4  5,9  6,3  5,5  6,7 |
| IC | 5,90  6,10  6,20  5,83  6,00  6,30 | 5,8  6,0  6,4  4,9  5,5  4,4 | 2,2  2,0  1,7  2,3  1,9  2,1 | 2,0  1,9  1,4  1,8  1,7  1,9 | 1,5  1,4  1,1  1,6  1,3  1,5 | -  -  -  -  -  - |
| O CHQ | 6,0  6,7  4,4  6,6  5,2  5,5 | 5,2  4,7  5,5  6,4  5,6  5,7 | 2,9  2,6  2,9  2,8  3,1  2,0 | 3,2  3,0  3,8  3,2  4,0  2,7 | 2,6  3,6  2,6  3,2  2,9  2,6 | 3,8  5,0  4,0  5,5  3,9  4,2 |
| O OA | 6,1  5,9  6,7  6,0  7,1  6,5 | 5,1  4,6  5,9  5,8  5,6  6,0 | 2,6  2,9  2,2  2,3  3,3  3,1 | 4,5  4,9  4,5  5,3  7,2  5,7 | 5,6  5,0  6,3  4,4  5,2  4,5 | 5,6  5,5  4,8  6,3  6,6  5,7 |
| TD OA | 6,1  4,9  5,6  5,4  6,6  6,0 | 5,5  4,8  6,0  5,8  6,1  6,3 | 5,5  4,8  6,0  5,8  6,1  6,3 | 5,3  6,2  6,0  5,5  5,1  4,9 | 6,0  6,3  5,9  6,6  4,9  6,6 | 5,9  6,2  7,1  6,0  5,8  5,9 |
| TD CHQ-OA | 5,8  6,5  5,6  7,7  5,0  4,9 | 5,4  5,7  5,4  5,6  6,0  5,4 | 2,2  2,0  3,3  2,5  3,1  3,6 | 4,3  4,7  5,1  4,4  4,4  4,0 | 4,2  6,3  4,9  5,7  5,5  4,9 | 6,1  5,3  4,9  5,0  6,2  5,5 |

NIC- Non-infected control

IC- Infected control

NI- NI infected

I- Infected

O CHQ- Orally administered chloroquine

O OA- Orally administered oleanolic acid

TD OA- Transdermally administered oleanolic acid

TD CHQ-OA- Transdermally administered chloroquine-oleanolic acid combination
