# Supplementary material for: The Effects of Transdermally Delivered Oleanolic Acid on Malaria Parasites and Blood Glucose Homeostasis in P. berghei-Infected Male Sprague-Dawley Rats
Source: PLoS One. 2016 Dec 1;11(12):e0167132. doi: 10.1371/journal.pone.0167132 (PMC5132303; doi:10.1371/journal.pone.0167132)
Supplement: S4 Table — IC- Infected control; O CHQ- Orally administered chloroquine; O OA- Orally administered oleanolic acid; TD OA- Transdermally administered oleanolic acid; TD CHQ-OA- Transdermally administered chloroquine-oleanolic acid combination. (DOCX) [file pone.0167132.s004.docx]

**Table 4: Terminal plasma insulin concentrations following a 4h oral glucose tolerance tests**

| **Controls** | | **CHQ** | | **O OA** | | **TD OA** | | **TD CHQ-OA** | |
| --- | --- | --- | --- | --- | --- | --- | --- | --- | --- |
| NI | IC | NI | I | NI | I | NI | I | NI | I |
| 11.000 | 9.000 | 18.510 | 19.001 | 7.062 | 9.558 | 8.766 | 10.018 | 10.987 | 13.987 |
| 9.970 | 9.600 | 18.990 | 22.049 | 8.010 | 8.864 | 8.864 | 7.645 | 8.766 | 10.766 |
| 12.000 | 9.900 | 20.901 | 15.613 | 6.080 | 12.467 | 8.815 | 8.310 | 10.305 | 13.805 |
| 11.001 | 11.200 | 19.970 | 20.014 | 8.400 | 11.020 | 9.220 | 10.011 | 11.010 | 11.990 |
| 11.010 | 10.110 | 16.701 | 19.621 | 9.031 | 9.490 | 10.010 | 9.010 | 9.009 | 11.971 |
| 10.790 | 11.190 | 16.718 | 21.331 | 10.000 | 10.590 | 9.000 | 8.025 | 10.010 | 11.880 |

IC- Infected control

NI- NI infected

I- Infected

O CHQ- Orally administered chloroquine

O OA- Orally administered oleanolic acid

TD OA- Transdermally administered oleanolic acid

TD CHQ-OA- Transdermally administered chloroquine-oleanolic acid combination
