# Supplementary material for: The Effects of Transdermally Delivered Oleanolic Acid on Malaria Parasites and Blood Glucose Homeostasis in P. berghei-Infected Male Sprague-Dawley Rats
Source: PLoS One. 2016 Dec 1;11(12):e0167132. doi: 10.1371/journal.pone.0167132 (PMC5132303; doi:10.1371/journal.pone.0167132)
Supplement: S5 Table — IC- Infected control; O CHQ- Orally administered chloroquine; O OA- Orally administered oleanolic acid; TD OA- Transdermally administered oleanolic acid; TD CHQ-OA- Transdermally administered chloroquine-oleanolic acid combination. (DOCX) [file pone.0167132.s005.docx]

**Table 5: Short-term effects of various treatments on plasma insulin concentrations of non-infected and infected animals**

| **NIC** | | | | | **IC** | | | | **O CHQ** | | | |
| --- | --- | --- | --- | --- | --- | --- | --- | --- | --- | --- | --- | --- |
| Day 0 | Day 7 | Day 9 | Day 12 | Day  21 | Day 0 | Day 7 | Day 9 | Day  12 | Day 0 | Day 9 | Day 12 | Day 21 |
| 10,201 | 10,090 | 11,702 | 11,702 | 9,990 | 9,910 | 11,000 | 11,900 | 10,690 | 9,990 | 29,120 | 30,330 | 16,001 |
| 11,000 | 9,900 | 10,930 | 10,930 | 10,637 | 11,310 | 11,363 | 9,990 | 11,551 | 10,210 | 30,110 | 32,010 | 17,010 |
| 10,330 | 11,318 | 10,110 | 10,110 | 9,590 | 11,000 | 11,017 | 12,011 | 11,770 | 10,111 | 27,110 | 26,990 | 16,010 |
| 9,661 | 10,150 | 11,130 | 11,130 | 10,010 | 10,510 | 10,218 | 10,530 | 12,010 | 11,417 | 29,190 | 29,600 | 15,333 |
| 11,001 | 9,200 | 8,965 | 8,965 | 10,330 | 10,011 | 11,111 | 11,010 | 9,839 | 9,800 | 26,600 | 26,870 | 16,661 |
| 10,111 | 9,362 | 10,171 | 10,171 | 9,830 | 9,717 | 11,441 | 11,131 | 10,010 | 8,977 | 26,012 | 28,318 | 15,110 |
|  | | | | | | | | | | | | |
| **O OA** | | | | **TD OA** | | | | **TD CHQ-OA** | | | | |
| **Day 0** | Day 9 | Day 12 | Day 21 | Day 0 | Day 9 | Day 12 | Day 21 | Day 0 | Day 9 | Day 12 | Day 21 | |
| 10,331 | 11,500 | 12,020 | 13,999 | 11,430 | 9,883 | 10,011 | 11,600 | 11,000 | 11,970 | 20,010 | 10,100 | |
| 10,048 | 9,610 | 12,990 | 12,890 | 9,770 | 11,580 | 9,580 | 11,011 | 10,770 | 10,880 | 20,930 | 12,113 | |
| 8,880 | 10,010 | 12,444 | 12,970 | 9,749 | 9,409 | 8,111 | 10,110 | 11,102 | 10,729 | 17,610 | 10,660 | |
| 11,790 | 10,203 | 12,877 | 11,884 | 8,930 | 10,050 | 9,371 | 12,030 | 10,021 | 9,991 | 15,969 | 9,593 | |
| 10,000 | 11,010 | 13,911 | 12,970 | 10,220 | 10,110 | 10,010 | 10,140 | 9,930 | 12,010 | 18,770 | 11,200 | |
| 10,220 | 10,380 | 11,990 | 12,608 | 11,110 | 11,600 | 9,250 | 11,281 | 11,381 | 8,990 | 18,330 | 10,010 | |

NIC- Non-infected control

IC- Infected control

NI- NI infected

I- Infected

O CHQ- Orally administered chloroquine

O OA- Orally administered oleanolic acid

TD OA- Transdermally administered oleanolic acid

TD CHQ-OA- Transdermally administered chloroquine-oleanolic acid combination
