# Supplementary material for: The Effects of Transdermally Delivered Oleanolic Acid on Malaria Parasites and Blood Glucose Homeostasis in P. berghei-Infected Male Sprague-Dawley Rats
Source: PLoS One. 2016 Dec 1;11(12):e0167132. doi: 10.1371/journal.pone.0167132 (PMC5132303; doi:10.1371/journal.pone.0167132)
Supplement: S6 Table — IC- Infected control; O CHQ- Orally administered chloroquine; O OA- Orally administered oleanolic acid; TD OA- Transdermally administered oleanolic acid; TD CHQ-OA- Transdermally administered chloroquine-oleanolic acid combination. (DOCX) [file pone.0167132.s006.docx]

**Table 6: Liver** **glycogen concentrations following oral administration of CHQ (O CHQ), OA (O OA) and transdermal application of OA (TD OA) and or CHQ-OA (TD CHQ-OA)**

| **Liver** | | | | | | | | | | | | | | | | | | |
| --- | --- | --- | --- | --- | --- | --- | --- | --- | --- | --- | --- | --- | --- | --- | --- | --- | --- | --- |
| **NIC** | | | **IC** | | | | **O CHQ** | | | **O OA** | | | **TD OA** | | | **TD CHQ-OA** | | |
| Day 9 | Day 12 | Day 21 | Day 9 | Day 12 | Day 21 | IC | Day 9 | Day 12 | Day 21 | Day 9 | Day 12 | Day 21 | Day 0 | Day 9 | Day 12 | Day 9 | Day 12 | Day 21 |
| 21,320 | 30,401 | 27,701 | 17,011 | 14,931 | - | 17,230 | 52,010 | 55,010 | 29,530 | 24,822 | 19,010 | 22,000 | 30,002 | 26,030 | 20,190 | 25,380 | 31,010 | 25,003 |
| 26,550 | 24,701 | 28,993 | 15,660 | 15,110 | - | 19,001 | 47,800 | 48,883 | 25,921 | 21,883 | 23,001 | 19,660 | 27,110 | 29,480 | 18,113 | 23,660 | 28,303 | 23,310 |
| 24,971 | 25,413 | 28,770 | 18,211 | 16,441 | - | 16,101 | 45,791 | 53,442 | 26,861 | 24,080 | 19,751 | 20,061 | 27,110 | 27,971 | 21,230 | 25,001 | 29,690 | 22,502 |
| 26,300 | 26,110 | 24,631 | 17,442 | 17,021 | - | 16,360 | 47,022 | 49,880 | 24,750 | 19,690 | 23,110 | 19,519 | 29,570 | 25,255 | 18,103 | 24,441 | 28,301 | 21,318 |
| 25,491 | 26,553 | 25,700 | 17,910 | 16,571 | - | 18,022 | 46,552 | 52,901 | 26,710 | 20,649 | 24,330 | 22,011 | 28,120 | 28,330 | 18,390 | 26,201 | 26,291 | 22,011 |
| 22,950 | 24,831 | 28,610 | 15,851 | 15,920 | - | 17,130 | 50,044 | 48,952 | 27,099 | 24,977 | 18,557 | 18,339 | 26,777 | 31,000 | 19,283 | 23,500 | 28,011 | 24,111 |

**Table 7: Muscle glycogen concentrations following oral administration of CHQ (O CHQ), OA (O OA) and transdermal application of OA (TD OA) and or CHQ-OA (TD CHQ-OA)**

|  | | | **Controls** | | | | **O CHQ** | | | **O OA** | | | **TD OA** | | | **TD CHQ-OA** | | |
| --- | --- | --- | --- | --- | --- | --- | --- | --- | --- | --- | --- | --- | --- | --- | --- | --- | --- | --- |
| **Day 9** | **Day 12** | **Day 21** | **Day 9** | **Day 12** | **Day 21** | **IC** | **Day 9** | **Day 12** | **Day 21** | **Day 9** | **Day 12** | **Day 21** | **Day 9** | **Day 12** | **Day 21** | **Day 9** | **Day 12** | **Day 21** |
| 51,60 | 52,110 | 53,991 | 53,901 | 52,371 | - | 52,829 | 60,770 | 64,329 | 56,002 | 51,790 | 40,721 | 56,044 | 57,330 | 44,170 | 54,600 | 50,893 | 41,351 | 46,971 |
| 55,010 | 49,663 | 56,701 | 52,441 | 49,910 | - | 51,430 | 63,881 | 67,092 | 54,907 | 52,011 | 39,277 | 54,770 | 54,709 | 41,481 | 53,595 | 52,444 | 41,208 | 43,010 |
| 51,000 | 55,331 | 50,710 | 53,410 | 51,111 | - | 52,500 | 63,960 | 65,770 | 55,910 | 47,273 | 37,190 | 54,744 | 56,630 | 41,795 | 52,717 | 49,770 | 40,440 | 42,770 |
| 52,401 | 52,970 | 54,311 | 55.001 | 50,051 | - | 48,911 | 62,891 | 63,701 | 55,810 | 48,700 | 40,110 | 55,712 | 54,979 | 44,101 | 56,011 | 50,320 | 39,101 | 46,066 |
| 48,500 | 52.801 | 50,771 | 49,881 | 52,501 | - | 53,220 | 62,791 | 64,019 | 53,220 | 50,011 | 39,361 | 53,576 | 51,920 | 41,551 | 52,220 | 52,559 | 41,117 | 43,161 |
| 53,731 | 55.011 | 53.910 | 50,201 | 49,811 | - | 51,972 | 63,797 | 66,333 | 54,825 | 46,699 | 37,339 | 55,190 | 56,810 | 40,698 | 55,001 | 51,869 | 38,509 | 46,201 |

NIC- Non-infected control

IC- Infected control

NI- NI infected

I- Infected

O CHQ- Orally administered chloroquine

O OA- Orally administered oleanolic acid

TD OA- Transdermally administered oleanolic acid

TD CHQ-OA- Transdermally administered chloroquine-oleanolic acid combination
